# Is one-stage f-URS without prior stenting really safe for solitary kidney patients with 1–3 cm renal stones?

**DOI:** 10.1080/0886022X.2021.1903929

**Published:** 2021-03-29

**Authors:** Yuzhuo Li, Qiqi He

**Affiliations:** Department of Urology, Key laboratory of disease of Urological systems, Gansu Nepho-Urological clinical Center, Lanzhou University Second Hospital, Lanzhou, Gansu, China

Dear Editor,

We read with interest Dr. Pan and his colleagues’ recent article published in Renal Failure: ‘The feasibility of one-stage flexible ureteroscopy lithotripsy in solitary kidney patients with 1–3 cm renal stones and risk factors of renal function changes’ [1]. The article described a meticulous retrospective study and provided useful information on glomerular filtration rate (GFR) changes in solitary kidney patients with large stones after flexible ureteroscopy lithotripsy (f-URS). However, the conclusion of the article ‘one-stage f-URS without prior stenting (PS) could be feasible for 10–30 mm renal stones in solitary kidney patients’ should be strictly reviewed before application in clinical practice based on the following issues.

1. Protecting residual nephrons should be the primary goal of surgery in solitary kidney cases. As reported by Pan et al. and other studies [2,3], shorter duration of surgery might be beneficial to protect renal function. Surgery duration of f-URS is mainly related to the stone burden (stone diameters, HU value, etc.) [3]. Hence, it is not recommended to perform one stage f-URS without PS in solitary kidney cases with large (>2 cm) stone burden since the surgery duration is longer, which could cause complications and loss of renal function in long-term.

2. A large sample size randomized control trial suggested that routine ureteral stenting is not mandatory for uncomplicated cases [4]. However, PS makes it easier to place the ureteral access sheath (UAS), thereby reducing the f-URS surgery duration [5]. A recent multi-center clinical study [6] with data of 724 patients showed that ‘ureteral stenting did not affect operative outcomes (stone-free rate), and increased the success rate of UAS placement (93.8% vs. 85.3%)’. The advantages of using UAS were：① regular drainage to maintain low intrarenal pressure; ② protecting the ureter; ③ expediting stone extraction [7]; ④ potentially enlarging ureteral diameters (clinical experience but no evidence in the literature, which might be due to lack of methodology for measurement). All these factors were regarded as favorable factors to reduce the surgery duration and improve the procedural safety. Therefore, for uncertain solitary kidney stone cases, PS and UAS should be applied to reduce the potential risks in f-URS.

3. One stage f-URS is widely utilized in common cases (not solitary kidney cases) since single use flexible ureteroscopy (F7.5-F8.7) and thin UAS (F10-12/F11-13) are available in China [8]. However, in uncertain ureteral conditions (perhaps narrow or tortuous), chronic kidney diseases (CKD) might exist in solitary kidney stone patients. Hence, PS and UAS placement are necessary in our view. For large stone burdens in solitary kidney cases, besides staged f-URS [3], the vacuum-assisted UAS combined with an intrarenal pressure monitor could prolong the surgery duration and improve surgical efficiency with little risks [9].

Based on the above evidence, we suggest that one stage f-URS without PS should not be recommended for solitary kidney patients, particularly with large (2–3 cm) stone burden. Given the potential life-threatening risks, further large prospective randomized studies are almost impossible to conduct on this issue due to ethical reasons. While we appreciate the meticulous efforts of all the authors who contributed to this study, it is important to note that the unreliable conclusions might harm the potential interests of patients if applied by inexperienced physicians in the clinic.
